# Heterotopic ossification after patellar tendon repair in a man with trisomy 8 mosaicism: a case report and literature review

**DOI:** 10.1186/1752-1947-5-453

**Published:** 2011-09-12

**Authors:** Austin Chen, Samuel Chmell

**Affiliations:** 1 Department of Orthopedic Surgery, University of Illinois at Chicago, 835 S Wolcott Avenue, M/C 844 Chicago, IL 60612, USA

## Abstract

**Introduction:**

Heterotopic ossification is the abnormal formation of lamellar bone in soft tissue. Its presence jeopardizes functional outcome, impairs rehabilitation and increases costs due to subsequent surgical interventions.

**Case presentation:**

We present a case of a 32-year-old African-American man with trisomy 8 mosaicism who developed severe heterotopic ossification of his right extensor mechanism subsequent to repair of a patellar tendon rupture.

**Conclusion:**

To the best of our knowledge there are no prior reports of heterotopic ossification as a complication of patellar tendon repair. This case may suggest an association between trisomy 8 mosaicism and increased risk of heterotopic ossification.

## Introduction

Heterotopic ossification (HO) is most commonly associated with musculoskeletal trauma, central nervous system disorders or injuries, severe burns, and elective surgery such as total hip arthroplasty [1]. The clinical signs of HO include increased joint stiffness, limited range of motion, warmth, swelling and erythema. Although its etiology is still unclear, important contributing factors include hypercalcemia, tissue hypoxia, alterations in sympathetic nerve activity, prolonged immobilization and imbalance between parathyroid hormone and calcitonin [2]. The overexpression of bone morphogenetic proteins (BMPs), among other systemic and local factors, also appears to play an important role in the pathophysiology of HO [2]. HO occurs in 3-90% of lower limb joint replacement cases, though only 3-7% is clinically significant based on the Brooker Classification of HO (Grades 3 and 4) [3,4]. HO can also be hereditary; similar to fibrodysplasia ossificans progressiva, progressive osseous heteroplasia, and Albright's hereditary osteodystrophy [3].

Complete somatic trisomy 8 is rarely compatible with life and often results in miscarriage [5]. Trisomy 8 mosaicism (T8M), on the other hand, is a form of trisomy 8 in which some of the body's cells have three copies of chromosome 8 while other cells still possess the normal two copies. T8M is an uncommon diagnosis affecting only one in every 25,000-50,000 live births. The timing and particular cell lineages in which nondisjunction occurs determine which tissues and cells are affected. Therefore, T8M can present with a wide range of clinical manifestations and extremely variable phenotype [6]. Some of the common musculoskeletal features of T8M include joint contractures, long and narrow thorax with wide sloping ribs, hypoplastic glenoid cavities, symmetrical widening of the clavicles, abnormal sternum, narrow pelvis and hip dysplasia [5-8].

## Case presentation

**Figure 1:**
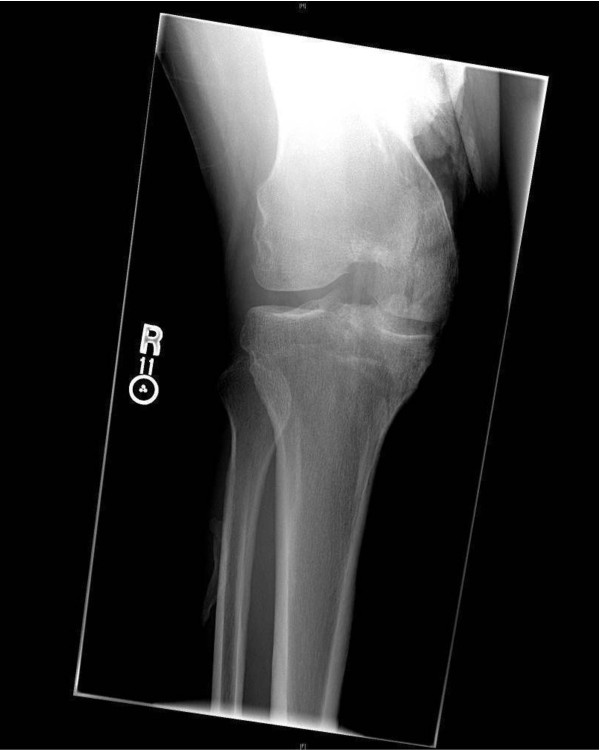
**Anteroposterior X-ray of the patient's right knee at the time of presentation**. Some HO is seen superior to his knee.

Our patient is a 32-year-old African-American man with a history of T8M syndrome documented by chromosomal analyses at an outside hospital. His syndrome is characterized by dysmorphic facial features including saddle nose deformity and a large forehead as well as mild mental retardation. He presented to our clinic with complaints of right knee pain and inability to completely extend his right knee after injuring it several months ago. On examination of his right knee he was able to achieve full extension passively but was unable to actively perform a straight leg raise. On palpation, there was generalized tenderness and a high riding patella with a palpable gap beneath it consistent with a patellar tendon rupture. X-rays revealed marked patella alta with some mild HO in his distal quadriceps musculature (Figure 1). Our patient was consented for right patellar tendon repair and possible excision of the HO.

During the operative repair, his patellar tendon was found to be avulsed off the inferior pole of his patella. A repair was accomplished by weaving sutures through the patellar tendon and drill holes in his patella. Postoperatively, our patient was placed in a long leg cast. Our patient was not given any therapy for HO prophylaxis.

Postoperative follow-up visits for the first six weeks revealed no obvious complications with proper wound healing and no complaints from our patient. At six weeks postoperatively, his cast was removed. Physical therapy was instituted at that time.

**Figure 2:**
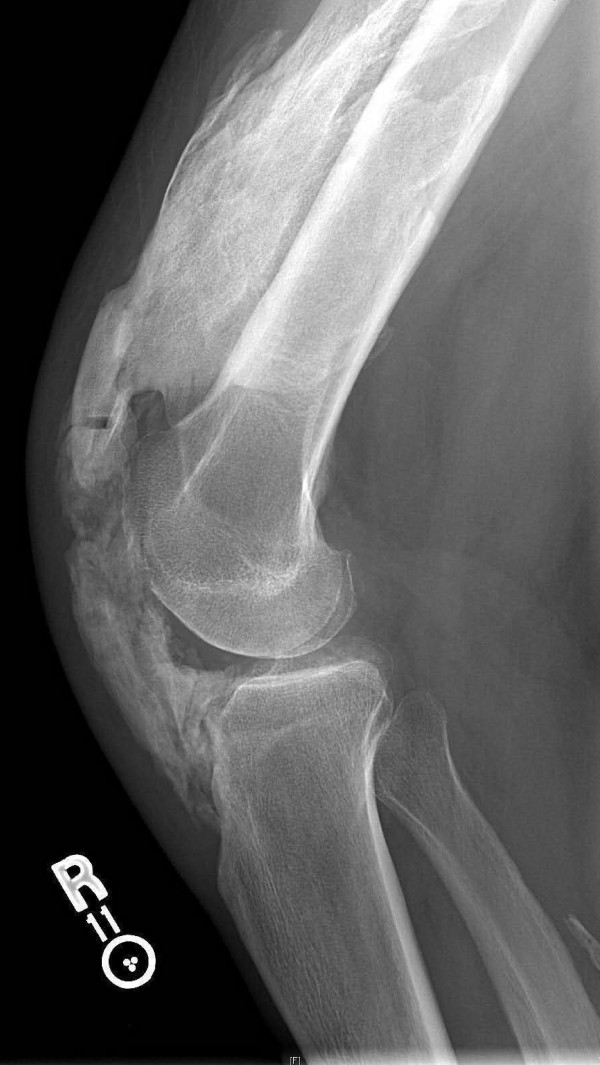
**Lateral X-ray three months after patellar tendon repair showing marked progression of HO**.

Follow-up visits for the next three months demonstrated a decreasing range of motion of his right knee. X-rays taken three months postoperatively revealed extensive HO within his quadriceps muscles as well as the patellar tendon (Figure 2). At four months postoperatively, our patient's knee was completely fused at 45 degrees. Despite the deteriorating range of motion, plantar and dorsiflexion remained intact. Sensation was intact and there was brisk capillary refill.

At this time our patient was given the option of leaving his knee locked at 45 degrees or performing a second surgery to fuse the knee in a more functional position. A total knee arthroplasty was not considered because our patient's quadriceps mechanism had ossified thereby eliminating active knee extension. After several additional opinions, our patient and his mother decided to proceed with a knee fusion.

**Figure 3:**
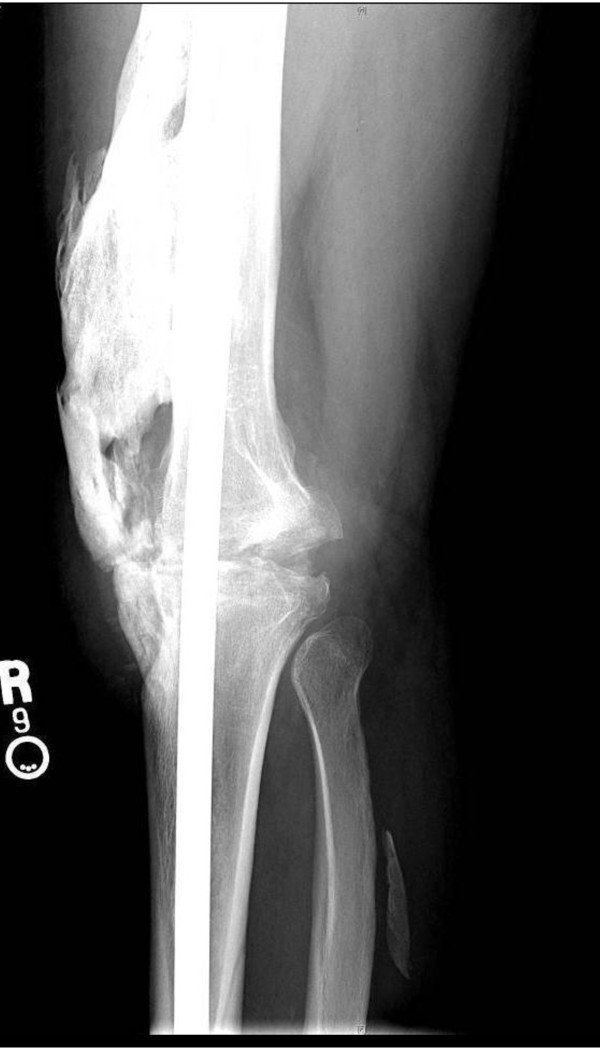
**Lateral X-ray of the patient's right knee after surgery to accomplish arthrodesis**.

A second surgical procedure was undertaken. Compression arthrodesis of the knee was accomplished with an intramedullary interlocking nail from the hip to ankle (Stryker T-2 Fusion Nail System) after the distal femur and proximal tibia were transversely denuded of cartilage and subchondral bone. Images taken after the surgery revealed a successful procedure (Figure 3).

## Discussion

After delaying treatment for several months for unclear reasons, our patient presented with mild HO on his initial radiographs. The subsequent trauma of the primary surgery to repair his patellar tendon was most likely a catalyst for the extensive additional HO that crippled his right knee mobilization. There are no documented cases of HO secondary to patellar tendon repair.

The aggressive nature of this patient's HO may be attributable to his T8M diagnosis. Chromosome 8 has been linked to certain BMPs. BMPs are part of the transforming growth factor beta (TGFβ) superfamily and play an important role in postnatal bone development [9]. BMP-1, located at 8p21, may explain the presence of abnormal bone formation in our patient with T8M [10]. BMP-1 has a unique structure and may play a role in activating other BMPs [10]. Extensive research is being conducted to better understand the biochemistry of these proteins.

Basic standards for HO prophylaxis have been relatively well established, but specifics are still debated. Current methods include non-steroidal anti-inflammatory drug (NSAID) treatment with indomethacin or localized radiation therapy. A recent study concluded that indomethacin is the gold standard for HO prophylaxis following total hip arthroplasty and, furthermore, is the only drug proven to be effective against HO following acetabular surgery [11]. Although radiation therapy has been shown to be slightly more costly than NSAIDs, other studies suggest that morbidities and quality of life differences associated with NSAIDs are difficult to quantify, and radiation therapy may remain the preferred prophylaxis of HO after total hip arthroplasty [11,12].

## Conclusion

It is our opinion that this patient's T8M status placed him at higher risk for developing HO postoperatively. There are no reports of HO as a complication of patellar tendon rupture or repair. A link between these pathological phenomena could explain the extensive HO in our patient and allow us to anticipate similar outcomes in T8M patients.

## Consent

Written informed consent was obtained from the patient's mother for publication of this case report and any accompanying images. A copy of the written consent is available for review by the Editor-in-Chief of this journal.

## Competing interests

The authors declare that they have no competing interests.

## Authors' contributions

AC participated as an observer in the case described, performed an extensive literature review and was primarily responsible for writing the manuscript. SC was the attending physician for the case described and provided guidance throughout the literature review and writing process. Both authors have read and approved the final manuscript.
